# Twinning and Individuation: An Appraisal of the Current Model and Ethical Implications

**DOI:** 10.3390/biology14020104

**Published:** 2025-01-21

**Authors:** Francis J. O’Keeffe, George L. Mendz

**Affiliations:** School of Medicine, The University of Notre Dame Australia, Sydney, NSW 2010, Australia; francis.okeeffe@nd.edu.au

**Keywords:** monozygotic twinning, totipotency, amniotic and chorionic arrangements, sesquizygotic twinning, ethics human embryo experimentation

## Abstract

Discourses on human embryo experimentation often refer to monozygotic twinning and individuation. A criterion to establish regulations that guide human embryo research proposes that individuation is achieved once the embryo ceases to have the potential for dividing into two or more viable entities at about 15 days of gestational age. This standard is based on an updated version of a developmental model initially proposed by George Corner. A fundamental problem with this approach is the model’s lack of sufficient evidence to explain adequately human embryo twinning and, consequently, to serve as a basis to establish appropriate ethical guidelines for embryo experimentation. In addition, subsequent formulations of Corner’s model added an extension of blastomere totipotency to different moments of gestation, without a proper scientific basis. The model is also challenged by monozygotic twinnings that result in placental and amniotic arrangements incompatible with Corner’s framework. Investigations into the physiology of fertilisation and of the zygote suggest that individuation may occur at a very early stage. An alternative description of monozygotic twinning may explain better sesquizygotic twinning events and serve to re-evaluate the individuation criterion. The study aims to investigate deficiencies in the embryology of this model and assess their ethical implications.

## Simple Summary

A legal disposition governing human embryo experimentation permits it to be conducted up to around 15 days of gestation. The foundation of this rule is the belief that at about this time, human embryos lose their potential to divide into two viable entities. Thus, it is not an individual and should not be granted the legal status that protects individuals from being subjects of many experimental procedures. The idea that human embryos have a twinning potential during their first two weeks of development originates with a hypothesis, advanced in 1955 by embryologist George Corner, that attempted to explain the arrangement of placentae and amnions observed in twin pregnancies. However, a body of evidence shows it is erroneous. Subsequent versions of Corner’s model extended to various moments of embryo development the ability of single cells to grow into full organisms, without a proper scientific basis. An alternative hypothesis to explain twinning from a single zygote is discussed in the light of embryo splitting that produced twins of different sexes. Results of investigations into human zygote maturing suggest that individuation may occur at a very early stage of development. The study discusses the ethical implications of using Corner’s incorrect model.

## 1. Introduction

Discussions of experimentation on human embryos have been informed by monozygotic twinning (MZT) models [1,2,3]. Individuation is regarded as a necessary criterion for human embryos to be considered individual human beings and, thus, afford them appropriate status [4,5,6]. It is argued that if an early human embryo can split into two or more distinct viable organisms, it is not a single individual; hence, there is no foundation to treat the embryo as an individual human being and to offer it protection until individuation is assured [7,8]. Advocates for early human embryo experimentation propose that individuation is guaranteed when the twinning potential of a zygote or embryo ceases. Often, this situation has been timed at about fifteen days post-fertilisation with the beginning of the primitive streak formation [9,10]. This understanding of MZT generation is used to justify a fourteen-day limit within which embryo research may take place [11,12].

A different view proposes that individuation is established at the generation of the zygote irrespective of any potential twinning [13,14,15]. This perspective considers the conditions of individuation to be identity and wholeness [16], which the zygote fulfils during its constitution and subsequent development as an embryo. If part of an embryo separated to become another human being, this would be a new individual [17], and the zygote would be a contingent whole.

The origin of the timing of MZT was a model presented by George Corner in 1955, which hypothesised that embryo splitting could occur at three possible gestational moments up to about fifteen days post-fertilisation [18]. This hypothesis suggested that an embryonic fragment may detach from a multicellular embryo and continue development to full gestation, and further proposed MZT potentiality up to the onset of gastrulation [18].

Subsequent representations of his model often attributed totipotency to blastomeres found in a multicellular embryo up to fourteen days post-fertilisation [19,20,21]. Totipotency (Lat. *totipotentia*, “capability for everything”) can be defined as the capacity of a single cell to develop into a complete organism [22], that is, the ability of a single cell to produce an individual [23].

Later descriptions of this model added the hypothesis that a human embryo could split within a fourteen-day period (or slightly beyond in the case of conjoined twins) leading to MZT [24,25,26,27]. However, several cases involving monozygotic twins question its validity [28,29,30,31]. In the absence of conclusive data, the cause of human MZT remains to be established [32,33].

Corner’s hypothesis also proposes that the timing of embryo splitting determines the number of placentae and amnions present in a multiple pregnancy. However, since its publication in 1955, there have been MZT reports with numbers of embryonic membranes inconsistent with his schema. These cases erode the validity of Corner’s model and necessitate a re-evaluation of the individuation criterion. This reassessment is required because individuation considered as established at fourteen days post-fertilisation is a basis for the enacted legislation that permits human embryo experimentation.

This investigation examines Corner’s MZT model and its subsequent formulations and appraises the biological conclusions that arise from accepting it as factual. The study discusses whether the model is supported by up-to-date human embryology data and documents an instance of atypical embryo MZT that produced twins of different sexes (sesquizygotic twins). Sesquizygosis occurs when individuals are generated from one egg fertilised by two separate sperm, resulting in twins that have a single maternal contribution [34]. A case involving sesquizygotic twins of different genders is reviewed owing to its potential to better explain when human individuation is established.

Frequently, ethical appraisals of human embryo experimentation have been based on representations of Corner’s model where totipotency is extended to moments of embryogenesis along a two-week timeline or even beyond [35,36]. Thus, it is appropriate to consider whether these proposals rely on contemporary embryological data and, then, appraise the ethical implications of the results of such inquiry. The concluding sections of this study propose that ethical determinations on the status of human embryos should not be made according to a hypothetical MZT model whose validity is questioned by a body of evidence.

## 2. Corner’s Model and Embryo Individuation

**Figure 1:**
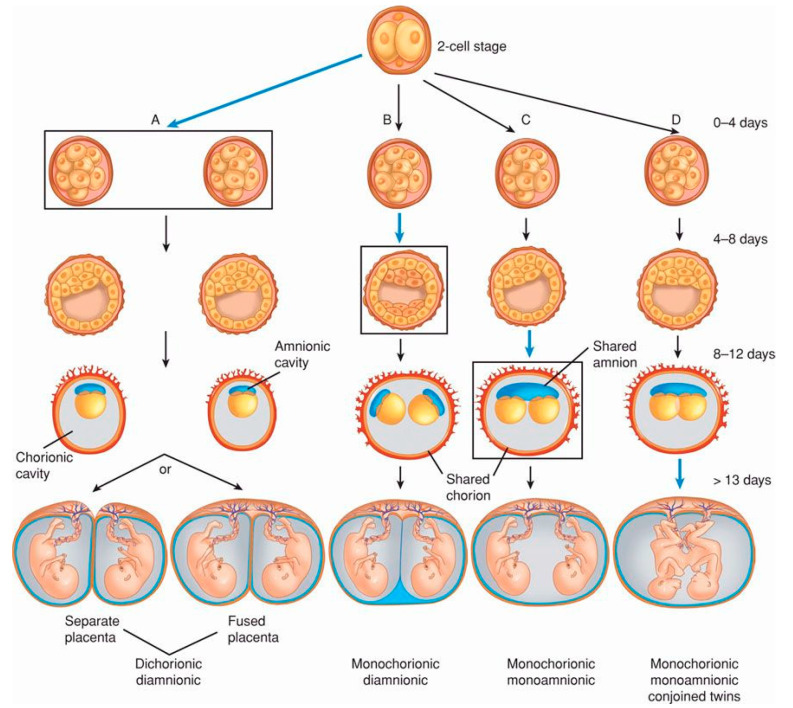
A contemporary representation of Corner’s model. (**A**) Dizygotic twin pregnancies are sustained by two placentae and two amnions. (**B**) Monozygotic twins. Early embryo splitting produces two placentae and two amnions. (**C**). Monozygotic twins. A splitting of the blastocyst generates twins sustained by a single placenta and two amnions. (**D**). Monozygotic twins. Late embryo splitting produces a single placenta and amnion. (From: https://obgynkey.com/multifetal-pregnancy-2/, accessed on 19 December 2024).

In 1955, Corner published an article that attempted to explain the generation of monozygotic twins. He put forward a “morphological theory of single-ovum twinning, tracing the various ways in which one egg might ultimately develop into two embryos” [18]. Human MZT is presented as a post-fertilisation event resulting from embryo division that may occur at one of three stages of early embryogenesis; namely, (i) the development of the first two blastomeres resulting from the first zygotic division, (ii) the separation of the blastocyst inner cell mass four to seven days after fertilisation, or (iii) the partition of the embryo at the onset of gastrulation (around day fifteen post-fertilisation) that produces two embryonic nodes. Also, the model speculated that twinning may occur at intermediate phases since development is continuous (Figure 1).

Corner’s hypothesis also attempted to explain configurations of chorions and amnions observed in multiple pregnancies. The chorion is an outer membrane enveloping the embryo and the placenta [37,38]; in a multiple pregnancy, “chorionicity” refers to the number of placentae present [31]. The amnion is an inner membrane enveloping the embryo and the placenta [37,38]; in a multiple pregnancy, “amnionicity” is the number of amnions that surround the embryos [39]. The model hypothesises that the number of chorions and amnions is determined by the stage at which embryo splitting may occur. Twins can be dichorionic and diamniotic (DC DA), monochorionic and diamniotic (MC DA), or monochorionic and monoamniotic (MC MA) [33]. Table 1 summarises the characteristics of Corner’s twinning model.

The model was modified in various developments that presented MZT as a possible embryo splitting occurring during the first fourteen-day period and slightly longer periods when conjoined twinning happens [24,27]. Table 2 provides a general outline of how Corner’s model is presently understood although variations exist among its contemporary representations.

Several conclusions follow from the modified model (i) dizygotic (DZ) twins are always DC; (ii) MC twins are monozygotic; and (iii) same-sex DC twins could result from fertilisation of one or more ova, but different-sex twins can only be dizygotic [40]. The hypotheses that MZT may occur during a fourteen-day period and that the cessation of possible embryo splitting is required for individuation led to the conclusion that the human embryo could not be considered an individual during its first two weeks of development.

The plausibility of the modified model(s) has been questioned multiple times on the basis that they remain undemonstrated in humans [40], and that the predicted connection between embryo splitting and chorionicity or amnionicity is not present in many observed cases [41]. Also, it has been argued that the models (i) are unverified by observations from in vitro fertilisation [30], (ii) are challenged by some documented instances of MZT [29], (iii) are inferential hypotheses accepted as factual without warrant [42], and (iv) lack sound biological foundations [43]. Thus, it is necessary to review the alignment between when MZT is proposed to occur and when current embryology considers totipotency to be present in the embryo.

## 3. Corner’s Model and Totipotency

An assessment of Corner’s model and its subsequent formulations requires a clear understanding of totipotency and pluripotency and ascertaining whether the model(s) align with current scientific data on the potentialities of embryonic cells. Totipotency applies to a cell beyond the zygote stage capable of initiating a full developmental program that will end in a complete organism [22]. It may also be understood as the ability of a single cell to differentiate into any cell or tissue type [37], albeit this is a questionable extension of its meaning. Thus, the one-cell zygote is totipotent in both senses [38]. Totipotency in early embryo cells is demonstrated when a single blastomere bisected from a multicellular embryo continues a developmental program up to full gestation.

Early in embryogenesis, the human embryo loses the totipotency characteristic of the zygote and comprises pluripotent cells that progress the developmental program. The complete ensemble of pluripotent cells can give rise to all the cell types that make up the body [44]. Partial ensembles of pluripotent cells might be capable of progressing embryogenesis to full development because they may inherit, dispersed amongst themselves, necessary elements of the developmental program required to proceed to a full organism. However, no single pluripotent cell detached from a multicellular mass can develop into a full organism. Thus, any attempt to explain MZT via detachment of a single pluripotent cell from a multicellular embryo is incorrect [45,46].

Corner referred to totipotency as “one of the fundamental questions of development, that of the potentiality for development possessed by the elements of a developing organism” [18] and suggested “three critical stages at which twinning might occur” [18]. He hypothesised that DC DA twinning might occur via the separation of totipotent human blastomeres at the first cleavage division [18]. Nonetheless, there appears no indication that he proposed later MZT to occur via fragmentation of a single totipotent blastomere. He wrote:

“In human development the inner cell mass is recognizable about day 4 after ovulation; implantation occurs at about day 7. If during this period some accident of development causes division of the inner cell mass, or starts the growth of two inner cell masses, then we have the beginning of twin embryos that will be enclosed in a single chorion” [18].

Corner suggested that an ensemble of detached cells might retain capacity to continue a developmental program that was initiated with the totipotent zygote. It remains indeterminate whether Corner considered totipotency necessary for MZT beyond the zygote. It is possible that Corner accepted the events depicted in subsequent formulations of his model, but this should not be inferred.

Notably, Corner extended MC MA twinning potential into the third week of embryogenesis up to the onset of gastrulation:

“The third variety of twinning, latest in time of origin (. . .) At this stage (about 15 days) the body of the embryo is patently present in the germ disc or ectodermal plate, and its location is determined as shown by the rudiments of the embryonic node and primitive streak, which are just appearing. In another day or two the embryo itself will become visible as the neural groove and folds appear. Duplication of the embryonic rudiment may occur at this stage if two embryonic nodes develop instead of one. Speaking in the technical language of general embryology, this is a process of double gastrulation” [18].

His model was subsequently modified and presented with an assumed extension of totipotency to human blastomeres up to and sometimes beyond fourteen days of embryogenesis [5,21,35,36,47,48,49]. In these later variations, MZT was assumed possible during the first fourteen-day period of human embryogenesis [24,27]. These modified conceptions of Corner’s hypothesis are a foundation for legislation permitting human embryo experimentation up to fourteen days post-fertilisation. Thus, the cogency of subsequent presentations of Corner’s model must be assessed.

## 4. Modifications of Corner’s Model

After the publication of Corner’s hypothesis, subsequent presentations of his model depicted totipotency extending to various gestational ages, which included the third and fourth cleavage divisions [19,20], the blastocyst stage [50], two weeks after fertilisation [21,49], or any blastomere from a two-to-three-week-old embryo [35,36,47,48]. Importantly, none of these studies presented scientific evidence to assert that totipotency could extend to these stages of human embryo development.

For example, a study published in 1970, asserted that totipotency could be collectively attributed to every blastomere that comprised a multicellular embryo, that is, to all cells of the human morula or blastocyst [51]. The bases for this assertion were experiments performed on lower organisms, not human embryos. No investigations involving mammals and blastomere totipotency were cited in this work.

Also, it was suggested that owing to “the potency of any cell up to gastrulation to become a complete entity, this particular zygote cannot necessarily be said to be the beginning of a specific, genetically unique individual human being” [35]. This line of thought prompted the assertion that:

“Those who want to object to embryo experimentation because it destroys a particular and identifiable human life would be on much safer ground were they to argue that a particular human life begins not at fertilization but at around day 14 after fertilization. By that time, totipotency has been lost” [21].

More recently it was proposed that at around the time of implantation, incorrectly considered to occur between days thirteen and sixteen post-fertilisation, human blastomeres lose their totipotency: “(The) primitive streak indicates that the embryo’s cells have become differentiated or restricted. In other words, the embryo’s cells lose their totipotency at this point; thereby precluding any further twinning (and/or subsequent recombination)” [49]. Once again, no evidence involving mammalian embryos was put forward.

Therefore, it is necessary to consider whether these studies have correctly timed the duration of human blastomere totipotency. Such an investigation will help clarify the moment at which human embryonic individuation is established.

For instance, during the 1960s, it was suggested that human individuation was not established until the MZT potential ceased [52].

In 1970, a study suggested that non-conjoined MZT was possible up to fourteen days and further proposed conjoined twinning potential beyond this point.

“It is well known that in this early stage of development the sphere of cells may split into identical parts to form identical twins. Twinning in the human may occur until the fourteenth day, when conjoined twins can still be produced” [1].

This became the most widely accepted modification of Corner’s hypothesis and led to the assertion that individuation is not assured until fourteen days post-fertilisation. No scientific data were provided that supported MZT up to fourteen days (and beyond with conjoined twins), and no explanation was given to alter Corner’s model. Thus, it must be considered whether scientific data exist to support the suggestion that blastomere totipotency in humans can extend up to and, possibly, beyond two weeks.

## 5. Blastomere Totipotency in Humans

Presently, there are no empirical data that indicate to which point in embryo development human blastomere totipotency extends because proof will require bisecting a blastomere from an embryo, transferring it to a woman’s uterus, and observing whether it develops into an entire offspring [23]. No instance has been reported of this occurrence. Nonetheless, inferences drawn from a small number of in vitro studies into human totipotency and investigations into nonhuman mammalian blastomere totipotency provide references to help understand when it ceases in early human embryogenesis.

Amongst the first endeavours to establish the extent of human blastomere totipotency involved an attempted cloning of cells extracted from seventeen genetically abnormal human embryos consisting of just a few cells each [53]. These embryos were divided into forty-eight single cells covered with a special gelatinous coating and placed in culture. It was alleged that a couple of cultures reached the thirty-two-cell stage of development. However, later this study was disregarded for its lack of scientific protocol, its complete methods and results were never published, and researchers were advised to discard their findings [54].

A subsequent investigation separated a single human blastomere from each of twenty-eight normally fertilised embryos that had reached the six-to-ten-cell stage at day three of embryogenesis and was allowed to develop to the blastocyst stage [55]. Only trophoblasts developed from fifteen (52.6 percent) of the blastomeres. The remainder were arrested at earlier stages and did not develop an inner cell mass (ICM) and trophectoderm (TE) [55]. These results indicated that human blastomeres at that developmental stage were not totipotent.

Another study involved human embryos at the two-to-five-cell and six-to-eight-cell stages that were split into two [56]. Sixteen of the twenty-four (66.6 percent) ensembles of blastomeres split from embryos at the six-to-eight-cell stage and five of twenty-six (19.2 percent) ensembles split from embryos at the two-to-five-cell stage developed into morulae or blastocysts [56]. These results did not provide evidence of totipotency because it can be assessed only by isolating single blastomeres; an embryo with several blastomeres excised may retain a capacity to develop into a blastocyst similar to that of the original morula. Importantly, the blastomere clusters derived from the splitting of human embryos exhibited no further growth beyond the blastocyst stage, indicative of an arrested developmental program.

An investigation split six four-cell stage human embryos into four isolated blastomeres that were cultured individually [57]. Most of the embryos derived from the blastomeres exhibited compaction and cavitation and, by day six, developed into small blastocysts with trophectoderm TE and embryoblast ICM. It was concluded that this development indicated totipotency. Later, the same group provided a more cautious appraisal when it suggested that human blastomeres derived from a four-cell embryo were only “potentially totipotent” [58]. In another investigation, sixteen blastomeres were bisected from four four-cell human embryos [58]. Two blastomeres produced developments from which human embryonic stem cell lines would be derived, indicative that at least one of the four-cell stage blastomeres was pluripotent [58]. These results tempered the previous assessment of totipotency, which was based on development that replicated the growth of a human blastocyst.

Caution must be exercised when appraising developmental behaviours that mimic the formation of a blastocyst as indicative of totipotency. Recently, there were reports of three-dimensional models of the human blastocyst, termed ‘induced human blastoids’ or ‘iBlastoids’ [59,60], defined as blastocyst-like, three-dimensional structures that recapitulate many of the events in the first ten days of development [61]. iBlastoids are created from somatic cells reprogrammed back to their pluripotent state. These cells are placed in microwells where they develop into a blastocyst-like 3D cellular structure to mimic a blastocyst. iBlastoids showed structural similarities to human blastocysts [61], but they mimicked only peri-implantation embryonic growth before developmental potential completely ceased, thus providing no insights into totipotency [59].

Discretion is necessary when considering events that resemble a gastrulating embryo as evidence of totipotency. Gastrulation comprises a series of developments post-implantation of the embryo that take place on its third week of life and includes the formation of the primitive streak and the emergence of the human body plan. In 2014, three-dimensional aggregates of embryonic stem cells called ‘gastruloids’ were reported [62] and described as a “multicellular in vitro model of a gastrulating embryo” [63]. Gastruloids exhibited some similarities to week three human embryos [64] but were found to skip stages of embryogenesis, did not display in full the morphology of early embryonic life [65], and lacked the capacity to develop structures required to support a pregnancy to full-term [66]. As discussed earlier, groups of blastomeres that show some developmental capacity provide no confirmation that either the ensemble can continue to full development or that a single cell of the group possesses totipotency.

## 6. Blastomere Totipotency in Nonhuman Mammalians

Attempts to understand human totipotency have been made with experiments involving nonhuman mammals. It is hypothesised that human totipotency might not differ significantly from that exhibited by other higher vertebrates. In fact, depending on the species, studies indicate that blastomere totipotency could extend to embryos at the two-, four-, or eight-cell stage [67]. In the mouse, totipotency has been demonstrated at the two-blastomere stage and remains to be demonstrated at the four-blastomere stage [68]. Totipotency was shown in blastomeres bisected from four- or eight-cell sheep embryos [69] in blastomeres derived from bovine embryos at the four-cell stage [70], and pigs were born from blastomeres isolated from an eight-blastomere embryo [71]. An attempt to create rhesus monkey MZ twins involved blastomeres separated from embryos at the two- or four-cell stage that grew in culture for six to eight days to blastocysts and were transferred to female monkeys [72]. A successful implantation was recorded, but the embryos derived from bisected blastocysts did not develop to full gestation for unknown reasons, which did not support their being totipotent. These results indicate that totipotency is unlikely to extend beyond the first few cleavage divisions in nonhuman mammalians.

## 7. Totipotency and Compaction

Presently, totipotency has been proven in some mammals only up to the eight-blastomere stage [15]. From earlier work, a consensus emerged from investigations into mammalians that totipotency plausibly would not extend beyond compaction [73,74]. Even if observations in nonmammalians were applicable to the human species, human totipotency beyond the first few cleavage divisions and past the point of compaction appears highly unlikely. A brief consideration of the timing of compaction in the human embryo will clarify possible limits of human blastomere totipotency potential.

An observation of 115 embryos [75] indicated that 99 (86.1 percent) embryos compacted between the four- and sixteen-cell stages, with initiation at the eight-cell stage being the most frequent (22.6 percent). Human morula compaction does not appear to take place at the same point in the development for all embryos, and a recent consensus situates compaction occurring around the eight-blastomere stage [37,38]. Thus, it seems that the first morphological cell differentiation at compaction could be the upper time limit of the loss of totipotency [75].

Contemporary discussions on totipotency include misunderstanding what it is. These errors can be summarised in four general misconceptions: (i) mistaking the ability of a cell to participate in a process of embryonic development such that it generates all the structures of the body; (ii) confusing the power of ensembles of early embryo cells to continue collectively to a full developmental sequence with the power of individual cells within the group; (iii) equating the expression of molecular markers characteristic of specific embryonic stages with the corresponding developmental capacities of the cells at those stages; and (iv) considering the ability of stem cells to replicate limited aspects of normal embryonic development as evidence of totipotency, or something very close to it [76].

Corner’s initial hypothesis is not disproven by investigations into the duration of totipotency, as is the case with later proposals that attributed totipotency to cells comprising a post-compaction human embryo. Nonetheless, the plausibility of Corner’s and later models depends on a proper understanding of the potentialities of human embryonic cells, and their validity depends on whether they correctly predict the arrangement of placentae and amnions in pregnancies with multiple embryos. Thus, the next section reviews documented cases of twinning with chorionicity and amnionicity inconsistent with Corner’s hypotheses.

## 8. Cases of Atypical Twinning

Corner’s model times the stages at which embryo splitting occurs by counting the number of amnions and placentae present in twin pregnancies. However, these descriptions for different amniotic and placental configurations do not explain several twinning cases.

### 8.1. DC DA Twins

Corner proposed that DC DA twins result from either DZ fertilisation or the spontaneous separation of two blastomeres following the first cleavage division [18]. Later formulations of the model present DC DA twins resulting from embryo splitting at any time prior to compaction [25,27].

It is difficult to observe whether the spontaneous separation of blastomeres occurs after zygote division; thus, the relationship between embryo splitting prior to compaction and DC DA twins has not been established [77]. Several case studies recorded MC fetal arrangement in DZ twins, including a review that documented thirty-one DZ MC twins between 2000 and 2017 [78]. These data challenge Corner’s view that DZ twins are always DC. Dizygotic MC twins are more prevalent in vitro fertilization-generated babies than those conceived in utero [79], prompting the consideration that unspecific factors in IVF are responsible for documented DZ MC fetal arrangements and not the timing of a splitting event [80].

Monozygotic quadruplets could be explained according to Corner’s model by two splitting events, the first of which is assumed to occur pre-compaction in the Fallopian tube, and the second one post-compaction [81]. The model proposes that an early splitting event determines DC, but this is at variance with observed instances of MC triplets and quadruplets and should prompt reconsideration of whether DC is determined by pre-compaction splitting [82,83,84].

### 8.2. MC DA Twins

Corner’s model and its subsequent modifications present MC DA twinning arising from either the splitting of the ICM or the spontaneous development of two embryoblasts [18]. Nevertheless, reported cases cast uncertainty on whether MC DA arrangement is always indicative of blastocyst fission.

Generally, MC DA placentation occurs in around 70 percent of natural MZT cases. In contrast, a recent study, documenting MZT cases over an eight-year period at a large IVF centre, found that close to 95 percent of recorded instances of MZT were MC DA [85]. If instances of MC DA are significantly more prevalent in embryos generated in vitro than naturally, then it could be hypothesised that unspecified factors present in IVF procedures and not simply the timing of splitting contribute to the likelihood of MC DA pregnancy, both in IVF and natural fertilisation.

Instances of the DC DA arrangement that resulted from a single human blastocyst transfer to the uterus have been documented [86,87], which would support the view that DC DA placentation is the result of blastocyst fission [88,89]. However, there is no empirical way to demonstrate that twinning had not already occurred when the assumed single embryo was transferred to the uterus; an inspection of the conceptus being transferred would only reveal a collection of cells within the pellucida prior to hatching. In addition, studies must explain the measures taken to avoid more than one embryo accidentally being transferred to the uterus. Zygosity of the transferred blastocyst must be confirmed empirically via genetic testing and not be merely assumed.

### 8.3. MC MA Twins

Corner hypothesised that the MC MA fetal arrangement occurs via embryo splitting at about day fifteen, or when conjoined twinning happens [18]. Posterior descriptions of his model depict MC MA placentation resulting from embryo splitting to occur after the beginning of the amniotic cavity formation in week two post-fertilisation or, in the case of conjoined twins, from incomplete embryo splitting after the beginning of the primitive streak at about day fifteen [90].

Extremely rare cases have been documented of the following: (i) MA triplets with two of the twins conjoined at the sacral area [91], (ii) non-MZ triplet pregnancies where three singletons are enveloped by one amnion [92], and (iii) non-MZ MA quintuplets [93]. According to Corner’s model, MA multiple pregnancies necessitate the occurrence of at least two splitting events, the first taking place at the earlier stages of embryonic development, to allow time for the second fission event to take place [90]. If a fragment of the original embryo undergoes a second splitting, then a triplet pregnancy could result; if both divided parts of the embryo split a second time, then quadruplets could form. This raises the question of how an MA triplet pregnancy could occur when there was an earlier first splitting of the human embryo. In Corner’s model and its subsequent developments, the mechanism to determine MA is a late splitting because to generate triplets or quadruplets would require an early division of the human embryo and a subsequent splitting, events that would yield a multiple amnion pregnancy.

Conjoined twinning is a rare phenomenon and is estimated to occur in about one in every 50,000–200,000 births [94]. A case of MC DA conjoined twins was reported twelve years after Corner’s proposal [95]. Since then, cases of DA conjoined twins who share part of a gastrointestinal system and abdominal wall have been documented [96,97,98,99]. Other cases include (i) triplets, two of whom were MC DA conjoined fetuses [100,101]; (ii) MC DA conjoined twins with discordant genotype for sex chromosomes [102]; and (iii) MC DA conjoined twins with body stalk, a rare severe defect of the body wall of twins [103]. Presently, there is no firm basis to consider conjoined twinning as a determining factor that establishes MC MA placental configuration.

Based on observed MC DA cases, experts have rejected Corner’s model and proposed alternative explanations of conjoined twinning generation [104,105]. Overall, in numerous cases, the observed arrangements of placentae and amnions do not follow the timing of MZT proposed by Corner’s model.

## 9. Sesquizygotic Twinning and Individuation

Sesquizygosis is an extremely rare third form of MZ twinning in which twins have single maternal contributions to their genome and share less than 100 percent of their paternal contribution [106]. Sesquizygosis occurs when two spermatozoa fertilise a single oocyte, resulting in an embryo comprising two genetically distinct types of cells.

A recent case of sesquizygotic twinning that resulted in twins of different sex has potential significance for understanding individuation [34].

In 2014, a woman at six weeks of gestation underwent ultrasound diagnosis confirming an MC DA twin pregnancy that suggested an identical twin pregnancy. However, at fourteen weeks, the ultrasound images revealed two fetuses of different sexes. Genetic tests showed each twin to be a chimera, that is, each twin had cells of two genetic types: one type with two X-chromosomes and the other with an X and Y chromosome [34]. Sesquizygosis was confirmed by a seventy-eight percent identical genome in both twins.

The similarity in the maternal contribution and differences in the paternal contribution to the twins’ genomes suggested dispermic fertilisation, with one sperm containing an X-chromosome, and the other a Y-chromosome. Typically, dispermic fertilisations produce an entity with three sets of chromosomes that would not initiate viable development.

**Figure 2:**
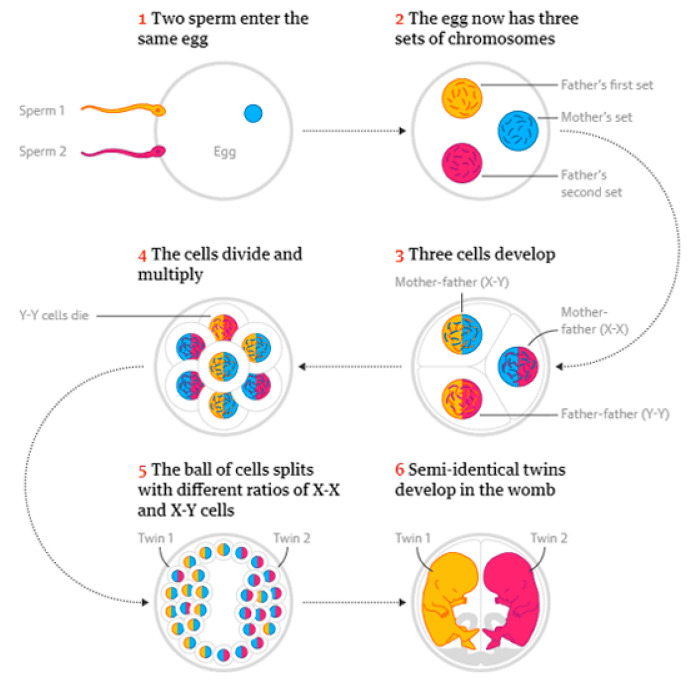
A hypothetical explanation of sesquizygosity. Following a dispermic penetration of an ovum, three zygotes form and only those with maternal contributions survive. The proliferation of the XX and XY zygotes results in two genetically different blastomere populations. Each population clusters closer to its original zygote producing a partial segregation of XX and XY cells that develop into chimeric female and male embryos, respectively. The maternal genetic contribution is represented in blue. The separate paternal genetic contributions are shown in red and orange. (From: https://gigazine.net/gsc_news/en/20190228-semi-identical-twins/, accessed on 19 December 2024).

A proposed explanation using Corner’s model was that the three sets of chromosomes derived from the ovum, X-sperm, and Y-sperm were able to sort themselves into three cells (Figure 2). Two had a biparental lineage, one was female (XX) and the other male (XY). The third cell was assumed to be of a uniparent (androgenetic) lineage and was outcompeted by the male and female blastomeres. It was hypothesised that a single human embryo comprising the genetically male and female cells underwent multiple cleavage divisions up until the blastocyst stage when embryo splitting would have taken place. This occurrence was described as “a twinning event [that] occurs around the blastocyst stage, as would normally occur in monochorionic diamniotic twins” [34]. In the context of Corner’s model, the presence of one placenta and two amnions suggested that embryo splitting occurred at this stage via the ICM division.

Importantly, it remained unexplained how the splitting of a blastocyst comprising male and female cells could produce twins of different sexes. Following Corner’s model, the ICM of the human embryo would be a multicellular mass comprising both male and female cells that divided in such a way that genetically female and male cells migrated to different sides of a blastocyst in different proportions by an unspecified force, following a blastomere segregation, mostly along the sex lineage, to yield twins of different sexes—a highly unlikely occurrence never observed in humans. Moreover, no driver was specified to affect the segregation of cells and how the process was sex-selective. Simply, Corner’s model is inadequate to account for blastocyst division resulting in different-sex twins who possess a seventy-eight percent identical genome.

A simpler explanation for the origin for the segregation of blastomeres is that two clusters of blastomeres developed, one from each of XX and XY zygotes. Successive divisions would keep blastomeres in each cluster close to its nearest neighbours and make them mostly clump together according to their origin, with limited mixing with the blastomeres arising from the other zygote. It could be argued that this uneven distribution of male and female cells remained after compaction since post-compaction cells largely maintain nearest neighbours. The partial segregation of blastomeres continued into the blastocyst stage prior to splitting, with each twin inheriting a preponderance of either male or female cells after separation. This explanation requires, at the morula stage of embryogenesis, a nonhomogenous distribution of XX and XY cells already existing within a single ICM of a sesquizygotic embryo, without explicating why and how this separation occurred.

In biology, a chimera is an organism composed of at least two genetically distinct types of cells [107]. In this instance of sesquizygosis, it could be proposed that chimerism occurred when admixtures of male and female cells segregated, accounting for the different ratios of male and female cells found in each sesquizygotic twin. If only segregation were responsible for chimerism, then the chimeric ratios would be consistent throughout the pregnancy; for example, if Twin 1 was 60/40, then Twin 2 must be 40/60 or if Twin 1 was 67/33, then Twin 2 would be 33/67. The fact that different ratios were observed at discrete times employing a variety of tests suggested that chimerism ratios were strongly affected by blood exchange. Monozygotic twins cannot become chimeric via twin-to-twin blood exchange because they have genetically identical cells, but cases of DZ MC chimeric twins have resulted from twin-to-twin transfusion [78,79,108,109,110]. Also, chimeric DZ MC twins of different genders with admixtures of XX and XY cells have been reported [111]. A probable explanation for the chimerism in this sesquizygotic pregnancy is that it occurred via placental anastomoses that enabled a bidirectional exchange of blood, as sometimes is observed in DZ MX twins, often referred to as “blood chimerism” [112].

Thus, a possible mechanism by which chimeric sesquizygotic twins of different sexes were generated could be that (i) fetal sex was established by atypical fertilisation that produced two totipotent cells of different gender, establishing individuation and generating male and female twins that incorporated some cells from the other twin; (ii) chimerism also resulted from shared circulation during gestation, as observed in some cases of chimeric DZ MC twins.

Objections to this hypothesis might include (i) the unlikelihood that two separate embryos could develop within the small space of the zona pellucida [8]; (ii) the possible aggregation of the two human embryos inside a single zona pellucida forming a single chimera, as observed in nonhuman animal studies [113]; (iii) the lack of verification of the existence of these events from in vitro observations in IVF [88].

In response, it needs to be considered that rare cases of monozygotic triplets and quadruplets have been explained proposing an initial early splitting that occurred inside the zona pellucida to produce two distinct embryos, followed by a later second splitting to establish either identical triplets or quadruplets [90]. Furthermore, it has been suggested that in MZT “a precocious action of proteinases may play a role in vivo, causing early weakening (or even partial dissolution) of the pellucida” [114], which would enable MZT to occur.

Experiments involving nonhuman animals that resulted in the creation of chimeras employed procedures completely alien to what naturally may occur during in vivo human fertilisation. The creation of mouse chimeras [113] has been cited as proof that two human embryos would necessarily aggregate to form a chimera inside a zona pellucida [15]. This mouse experiment used the compression of an oocyte during meiosis-I to prevent the normal release of a polar body and resulted in the symmetrical division of the ooplasm into two cells of similar size within the zona pellucida. The zona pellucida was then manipulated with laser techniques and both cells inside the pellucida were simultaneously fertilised. These data are inadequate to help explain the spontaneous generation of human MZT.

Although MZT has not been observed following fertilisation in IVF programs but during a splitting at a later stage [88], it should be highlighted that the rate of twinning increases for a single embryo when (i) it is frozen and subsequently thawed, (ii) it is matured for five to six days before transfer, and (iii) it undergoes assisted hatching [115]. The first two risk factors refer to embryo development within the zona pellucida. Spatial separation of embryos inside the zona pellucida may not be required, but a simple parallel development in clusters may be sufficient. In fact, a possible determinant of monochorionicity is a fusion of trophectoderm; thus, the developing embryos would only appear as a single cluster of cells during the early stages of embryogenesis.

## 10. Individuation and Polarity

Individuation could be discussed in the context of cell polarity of the human zygote or embryo. Polarity refers to “the tendency of living organisms or parts to develop with distinct anterior and posterior (or uppermost and lowermost) ends, or to grow or orientate in a particular direction” [116]. The presence of this asymmetry could be viewed as indicative of an individuated entity. An investigation into mice cell polarity proposed that these asymmetries are present in the zygote before cleavage begins [117]. Confirming these data, a study of polarity in mice embryos found that the first two blastomeres following zygotic cleavage division contribute independently to opposite poles of a mouse blastocyst [118]. It was concluded that the first two blastomeres possessed distinct ‘fates’ that made them different from the polarised zygote from which they developed [119], suggesting that they were not identical.

In response to these conclusions, it has been objected that polarity is only empirically verified at the blastocyst stage and that results suggesting that polarity is established at the constitution of the zygote are merely an artifact of laboratory techniques used [120]. However, more recent studies appear to verify that polarity is traced back to the earliest developmental stages in mammals [121]. A combination of polarity cues, cytoskeleton, and cell-to-cell communication interact and regulate the orientation of the early embryonic division planes [122].

Further evidence supporting zygotic individuation is provided by calcium ion movements observed in mouse fertilisation studies. Calcium ion waves ensure the events constituting egg activation occur in the correct temporal order and have possible long-term effects on both gene expression and individual development [123]. These calcium ion waves are initiated by fertilising sperm and terminate with pronucleus formation [123].

In humans, “The zygote is polarized because it has designed body axes and an asymmetric distribution of its elements” [124]. Thus, the first human zygotic division could be considered ordinarily as an asymmetric division of an already individuated entity [125]. An investigation of the self-organising properties of human embryos in the absence of maternal tissues found that the embryos exhibited development capacities slightly past the moment they would normally implant into the uterine lining [126]. These results indicated that early human embryos possess remarkable autonomy and self-organising capabilities without the support of the maternal host [126]. There appears to be a continuum of individuation starting with the polarized zygote and extending to the autonomous early embryo.

## 11. An Alternative Model of Twinning

A hypothetical model to explain sesquizygotic twinning proposes that MZT occurs at the first cleavage division of the zygote that, instead of producing two ordinary blastomeres, results in two totipotent cells with a zygote phenotype [41,43,77]. The model is hypothetical, but it provides a cogent explanation of sesquizygotic twinning and is relevant to the discussion on human embryonic individuation.

This hypothetical model is based on three considerations: (i) MZT occurs when two totipotent cells result from the cleavage of the zygote; (ii) chorionicity is determined by the fusion or non-fusion of the twins’ trophectoderm within the zona pellucida; and (iii) amnionicity depends on the distance separating each ICM of the MZT embryos.

The first consideration states that MZT is a variant of the constitution process (fertilisation) and not a self-construction event (gestation) of the human embryo [41]. Ordinarily, the constitution process concludes in the formation of one totipotent cell with a zygote phenotype [41]. For MZT to occur, a prolongation of fertilisation takes place and the first cleavage division, instead of resulting in two blastomeres, produces two genetically identical totipotent cells with a zygote phenotype [41], which develop within the zona pellucida that surrounds the plasma membrane of mammalian oocytes [43,77]. Different developmental clusters are established owing to the blastomeres generated by keeping close to their nearest neighbours.

This alternative model also suggests that prior to hatching, the fusion or non-fusion of trophectoderm determines chorionicity. If no fusion occurs, dichorionicity is established and the embryos continue to develop on separate chorions; if trophectoderm fusion occurs, monochorionicity ensues. This description closely mirrors Corner’s model in which dichorionicity is determined by the parallel development of the first two blastomeres. Notably, Corner once raised the possibility that if embryo splitting does not determine chorionicity, a “deceptive fusion of the membranes” might influence the placental arrangement [18]. Thus, according to this hypothesis, embryo splitting would have no relevance to chorionicity.

Finally, in this alternative hypothetical model, amnionicity depends on the distance between the ICM of each developing human embryo [43,77]. If ICM were near together, a common amnion could develop, and MC MA twins would be generated. If ICM were sufficiently apart, diamnionicity could result, producing MC DA twins. The observation of non-MZ MA quadruplets [93] requires scrutiny of this third hypothesis. The existence of these quintuplets does preclude that the distance between two ICM within a zona pellucida may determine MA in some instances, and suggests this may not be the sole factor determining MA.

## 12. Discussion of Ethical Issues

Legislations that permit human embryo experimentation up until day fourteen of embryogenesis are founded on Corner’s model and its subsequent developments. Thus, ethical assessments of human embryo experimentation must consider first the correctness of its biology.

This model was proposed with limited data on the duration of human blastomere potentialities, yet MZT capacity was extended into the third week of embryogenesis at the onset of gastrulation—a hypothesis without empirical support.

The present study does not suggest that totipotency is necessary for MZT to occur but clarifies that subsequent representations of Corner’s model erroneously attributed totipotency to cells that comprise a post-compaction multicellular human embryo, leading to the mistaken assertion that the early human embryo was a loose amalgamation of totipotent cells, any one of which may detach to establish MZT during the first fourteen days of embryogenesis.

Corner’s model was not proposed to discern the individuation of the human embryo; rather, it attempted to explain the configurations of placentae and amnions in multiple pregnancies. Yet, a significant body of evidence casts doubts upon its cogency.

Sesquizygotic twinning that produces different-sex twins cannot be coherently explained with Corner’s model and prompts consideration of other models to explain MZT. The alternative model outlined in this study is not proposed as proven and remains theoretical. It was reviewed because it provides a model for the generation of different-sex sesquizygotic twins, conceptualising that it may have occurred as a variation of a fertilisation event that established individuation.

A corollary from the sesquizygotic observations is that embryo twinning would not be considered a fission event whereby a single existing entity ceases to be and two new individuated embryos come into existence. Rather, it would be understood as a variation in fertilisation that concludes with the first zygotic cleavage. The ethical significance of this conclusion is that it eliminates MZT potential as a relevant consideration for individuation and ascertaining the status of the human embryo.

## 13. Conclusions

Caution should be exercised before accepting Corner’s model to establish the timing of human embryo individuation because it is based upon speculative biology that does not account for many observations. It remains a conceptually defective hypothesis and, thus, unsuited to serve as the basis for ethical determinations regarding the beginning of human life. The model has hindered proper discussions on embryo individuation, which must incorporate present-day discoveries and understanding of embryology.

Current embryology has set aside the misunderstanding that the early human embryo is a loose cluster of cells during the first 14 days of development. Rather, the early human embryo is a highly complex unified entity comprising multiple pluripotent cells whose function is orchestrated according to a program established with the constitution of the zygote.

Embryo splitting hypothesised to occur at three gestational moments was connected to different arrangements of chorions and amnions. This relationship is defective because (i) MZT is timed when it is unlikely that all parts of the human embryo have equal ability to develop to a full organism; (ii) there are documented MZT twinnings reporting chorionicity and amnionicity irreconcilable with his theory; and (iii) sesquizygotic twinning events are unexplained satisfactorily by this model. Human embryonic individuation cannot be ascertained based on this unproven model.

The data from current embryology should constitute the foundation for a revised appraisal of the status of the early embryo. Sesquizygotic twinnings open vistas for considering new MZT models and should inform future discussions on individuation. A better understanding of individuation is now possible because contemporary biology has revealed previously unknown facts about the human embryo, upon which sound bioethics can be established.

Notwithstanding the scientific data acquired to understand individuation, the ethical debate has been stifled by attributing excessive significance to MZT models. Individuation is a concept too complex to be limited within the parameters of speculative MZT hypotheses.

## Figures and Tables

**Table 1:** Corner’s model of twinning. DC DA: dichorionic diamniotic; MC DA: monochorionic diamniotic; MC MA: monochorionic monoamniotic.

Zygosity	Twins	Time of Split	Structure at Splitting	Chorions	Amnions
Dizygotic	DC DA	No split	No split	2	2
Monozygotic	DC DA	Day 1	Zygote	2	2
Monozygotic	MC DA	Days 5–7	Blastocyst	1	2
Monozygotic	MC MA	Day 15	Gastrula	1	1

**Table 2:** Contemporary representation of Corner’s model. Abbreviations for twin embryonic membranes are as in Table 1.

Zygosity	Twin Embryonic Membranes	Time of Split	Chorions	Amnions
Dizygotic	DC DA	No split	2	2
Monozygotic	DC DA	Days 1–3	2	2
Monozygotic	MC DA	Days 4–7	1	2
Monozygotic	MC MA	Days 8–14	1	1
Monozygotic	MC MA Conjoined	After the beginning of the primitive streak	1	1

## Data Availability

Data are contained within the article.
